# Genome-Wide Association Study and Marker Development for *Fusarium Oxysporum* Root Rot Resistance in Soybean

**DOI:** 10.3390/ijms252312573

**Published:** 2024-11-22

**Authors:** Yuhe Wang, Jinfeng Han, Xiangkun Meng, Maolin Sun, Shuo Qu, Yuanyuan Liu, Yongguang Li, Yuhang Zhan, Weili Teng, Haiyan Li, Xue Zhao, Yingpeng Han

**Affiliations:** Key Laboratory of Soybean Biology in Chinese Ministry of Education (Key Laboratory of Soybean Biology and Breeding/Genetics of Chinese Agriculture Ministry), College of Agriculture, Northeast Agricultural University, Harbin 150030, China; wangyuhe@neau.edu.cn (Y.W.); hjf272463@163.com (J.H.); 15647065179@163.com (X.M.); smaolin2022@163.com (M.S.); shuoquneau666@163.com (S.Q.); liuyuanyuan00516@163.com (Y.L.); yongguangli@neau.edu.cn (Y.L.); zyhsoybean@163.com (Y.Z.); twlneau@163.com (W.T.); lhy2112002@163.com (H.L.)

**Keywords:** soybean, *Fusarium oxysporum*, genome-wide association analysis, CAPS, KASP

## Abstract

*Fusarium oxysporum* root rot (FORR) is an important disease threatening soybean production. The development of marker-assisted selection (MAS) molecular markers will help accelerate the disease resistance breeding process and achieve the breeding goal of improving soybean disease resistance. This study evaluated the FORR disease resistance of 356 soybean germplasm accessions (SGAs) and screened resistance-related loci using genome-wide association analysis (GWAS) to develop molecular markers for MAS. A total of 1,355,930 high-quality SNPs were analyzed, 150 SNP sites significantly associated with FORR resistance were identified, and these sites were distributed within 41 QTLs. Additionally, 240 candidate genes were screened near these QTL regions, involving multiple functions such as hormone metabolism, signal transduction, stress defense, and growth regulation. Cleaved amplified polymorphic sequence (CAPS) and Kompetitive Allele-Specific PCR (KASP) molecular markers were developed based on candidate genes with significant SNP loci and beneficial haplotypes. The CAPS markers, S15_50486939-CAPS1 and S15_50452626-CAPS2, can effectively distinguish resistant and sensitive genotypes through enzyme digestion. The KASP marker is based on S07_19078765-G/T and exhibits a genotype clustering pattern consistent with disease resistance, demonstrating its application value in breeding. The CAPS and KASP markers developed in this study can provide reliable tools for MAS in FORR disease-resistant varieties. The research results will help reveal the genetic structure of FORR disease resistance and provide support for efficient breeding.

## 1. Introduction

Soybean root rot is one of the soil-borne fungal diseases that can occur at any growth stage of soybeans, eventually leading to soybean death and seriously reducing yield [1]. The main pathogens of soybean root rot include *Fusarium* spp., *Pythium* spp., *Phytophthora* spp. and *Rhizoctonia* spp. [2,3,4,5]. Additionally, in many regions, *Fusarium* is the predominant pathogen of soybean root rot. *Fusarium oxysporum* is one of the most common plant pathogens in soybean root rot. It can also cause vascular wilt in many plants. *F. oxysporum* has a wide host range and has caused serious losses to about 100 crops, including vegetables, flowers, field crops and cultivated crops [6,7,8]. The pathogen ranks fifth among the ten most economically important plant pathogens [9].

After the fungal spores of *F. oxysporum* colonize the soil, their hyphae can grow toward the plant and attach to the plant roots. The hyphae then grow along the roots and enter them via natural openings in the root system, such as wounds or where lateral roots emerge. Subsequently, hyphae grow and produce spores in the vascular system, causing blockage of the xylem. Eventually the above-ground parts of the plant begin to wilt due to a lack of water and nutrient supply. During the infection period of the plant host, *F. oxysporum* will continue to reproduce, thereby releasing new fungal spores, causing disease in other surrounding plants [10,11]. When soybean plants are infected by *F. oxysporum*, the lower taproot and lateral roots will turn brown to black, and obvious vascular discoloration and root rot will occur [12]. To maintain plant growth, secondary roots sometimes grow from the missing parts of the taproot. If root rot becomes severe, infected soybeans may develop foliar symptoms including chlorosis of the edges or entire leaves, stunting, wilting, and eventually leaf drop [12]. However, this pathogen is difficult to eradicate in the soil and has a wide host range, making it very difficult to control in plant production once it occurs. Although some chemical fungicides are effective against *F. oxysporum*, these chemicals are expensive and harmful to the environment. Seed treatment can be used to control *Fusarium* infection in the seedling stage. In the late stage of plant growth, the agent will lose its effectiveness and cannot fully control root rot. Moreover, long-term use of a single agent may lead to pathogen resistance. In summary, the wide host range and persistence of *F. oxysporum* in soil make it a major challenge in agricultural production.

Plant disease resistance is complex and affected by both genetic and environmental factors. The discovery of disease resistance-related genes is crucial for the selection and breeding of disease-resistant plants. In molecular breeding, genome-wide association studies (GWAS) have become a powerful method to establish the relationship between molecular markers (such as single nucleotide polymorphisms (SNPs)) and phenotypic traits. It can combine high-density and high-quality marker data with multiple phenotypic data to analyze loci related to disease resistance or stress resistance and obtain candidate genes related to phenotypes [13]. *F. oxysporum* resistance-related loci have been reported through GWAS in different plants, including Melon [14], *Arabidopsis* [15], and Banana [16]. In soybean, GWAS have successfully identified markers associated with a variety of disease resistance traits, including sudden death syndrome [17], *Sclerotinia* stem rot [18], and soybean mosaic virus [19]. In a research on soybean root rot, 279 soybean germplasms from the Yangtze-Huaizhou region were evaluated for resistance to *Phytophthora* root rot. Three candidate genes were identified by detecting strong peaks on chromosome 13 using GWAS [20]. The resistance of 314 soybean germplasms to *Fusarium graminearum* root rot (FGRR) was evaluated, and 12 loci and 9 candidate genes associated with FGRR resistance were found through GWAS [21]. A total of 248 soybean varieties from the USDA germplasm collection were evaluated for FGRR resistance, and six FGRR-associated genes were identified through GWAS [22]. However, there are few reports on GWAS mapping to investigate resistance to *Fusarium oxysporum* root rot (FORR) in soybeans. Only Sang et al. evaluated the resistance of 350 soybean germplasm resources to FORR and detected 8 SNP loci on chromosome 6 related to FORR through GWAS [23]. 

In the last decade, whole-genome SNP-derived CAPS and KASP molecular markers have emerged as potential genetic markers for uncovering the genetic regions affecting complex types of qualitative and quantitative traits in differentially derived populations of unexplored botanical groups. CAPS markers can be detected by PCR, restriction enzyme digestion, and agarose gel electrophoresis. CAPS markers are widely used in plant genetics and breeding research, especially in soybeans. For example, soybean drought tolerance [24], soybean mosaic virus [25], soybean cyst nematode (SCN) resistance [26], soybean seed Cadmium accumulation [27], soybean *Phytophthora sojae* resistance [28], soybean powdery mildew resistance [29].

In this study, we conducted a GWAS on soybean FORR resistance based on 356 SGAs and 1,355,930 SNPs. The purpose of this study was to identify SNP loci and candidate genes associated with FORR resistance through GWAS analysis. In addition, a genotyping method for SNP loci significantly associated with FORR resistance was established to provide a scientific basis for soybean disease resistance breeding efforts.

## 2. Results

### 2.1. Evaluation of Resistance to FORR in 356 Soybean Germplasm356 Soybean Germplasm Accessions

**Figure 1:**
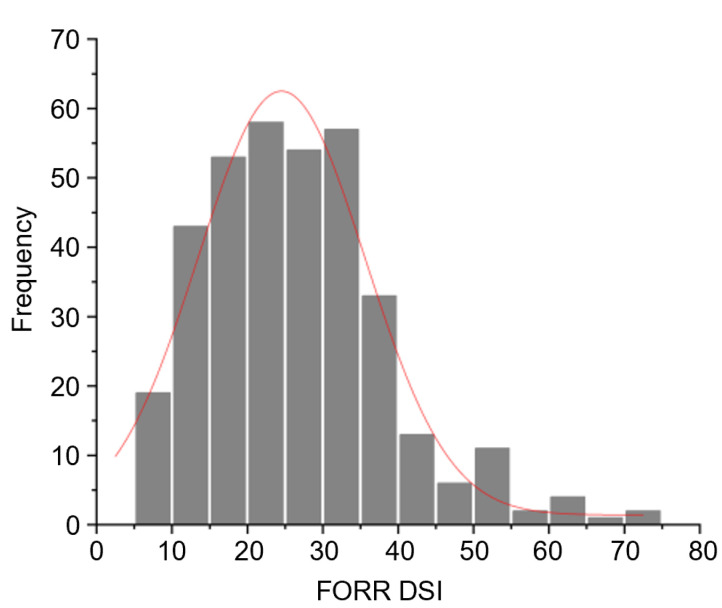
Disease severity index (DSI) of 356 SGAs exposed to FORR.

A total of 356 SGAs were inoculated with FORR, and we observed a wide phenotypic variation in SGAs in response to FORR as measured by the disease severity index (DSI) (DSI, refer to Supplementary Table S1). The DSI values ranged from 5.35 to 73.62, with an average value of 26.60. The distribution of DSI was continuous and unimodal, which was consistent with the characteristics of quantitative traits (Figure 1). This finding indicates that the natural soybean populations we utilized are suitable for GWAS analysis due to their abundant genetic variation. In the FORR resistance evaluation, the number of highly susceptible SGAs was the largest, accounting for 32% of the total SGAs. Only 5.3% of the total SGAs showed high resistance to FORR. We found no SGAs immune to FORR (Table 1).

### 2.2. Quality Control, Distribution of SNPS, and Linkage Disequilibrium Decay

**Figure 2:**
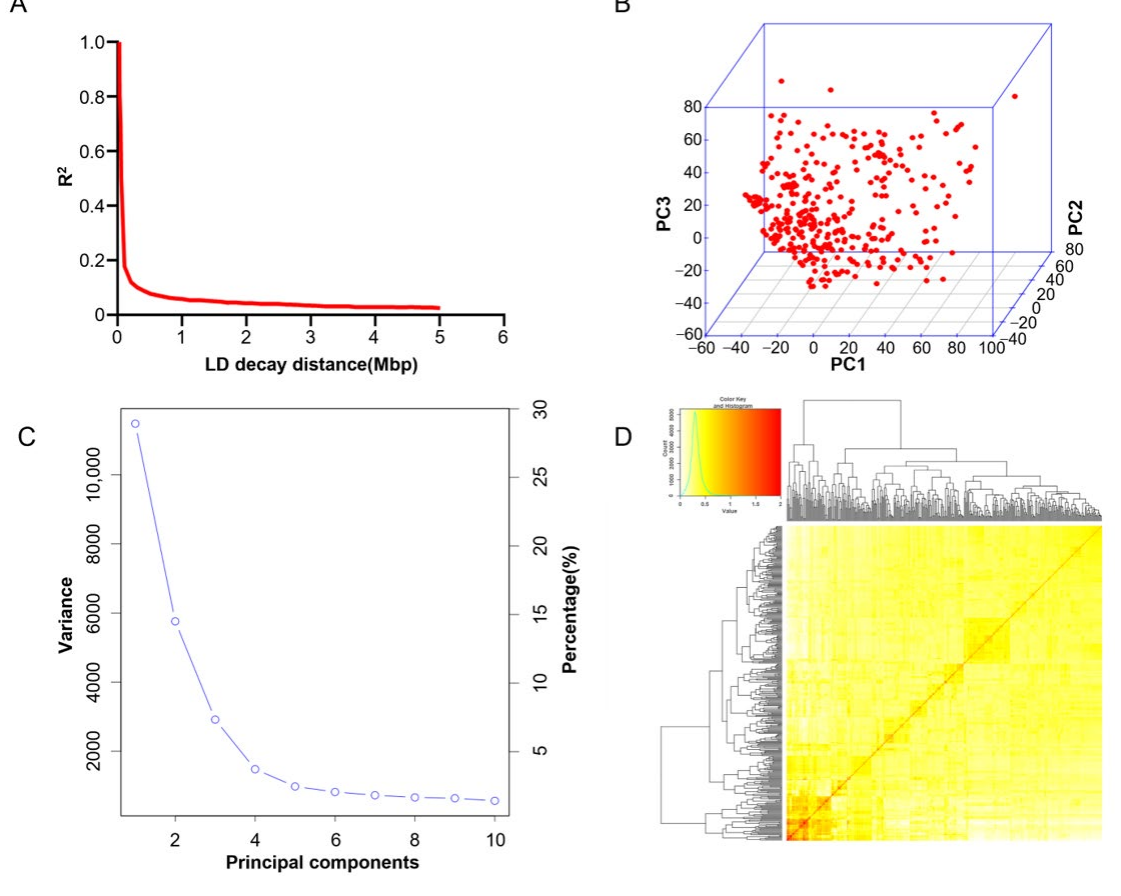
SNP distribution and mapping genetic data of populations. (**A**) Linkage disequilibrium (LD) decay of the genome-wide association study (GWAS) population. (**B**) Population structure of soybean germplasm collection reflected by principal components. (**C**) The first three principal components of the 1,355,930 SNPs used in the GWAS. (**D**) A heatmap of the kinship matrix of the 356 SGAs.

We performed genotyping using 356 SGAs resequencing data. A total of 1,355,930 high-quality SNP loci are distributed on 20 soybean chromosomes. These SNPs were distributed on 20 chromosomes of soybean. The average LD distance was 207 kbp, indicating that these soybean materials had high genetic diversity (Figure 2A). Principal component analysis and kinship analyses were performed using the entire SNP set to capture the overall population stratification of the association panel. The first three PCs contributed to 24.37% of the variation in the mapping population (Figure 2B,C). The soybean genetic relationship matrix showed that the level of relatedness among the 356 SGAs individuals used in this study was low (Figure 2D).

### 2.3. Genome-Wide Association Analysis and Candidate Gene Prediction Associated with FORR Resistance

**Figure 3:**
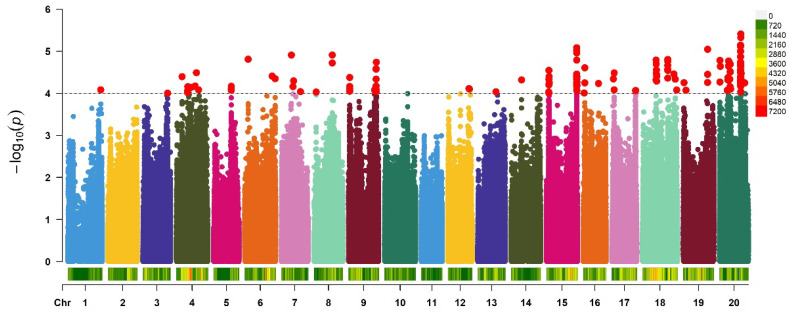
Manhattan plot of association mapping of FORR DSI in soybean. Different colors in the Manhattan plot represent SNPs from different chromosomes in soybean, the dashed line indicates the significance threshold (−log10(*p*) = 4.0). Each red dot above the threshold represents a SNP significantly associated with FORR.

We performed GWAS to identify significant SNPs associated with FORR. A MLM was used to identify association signals using the R package GAPIT. Each dot represents an individual SNP, the X-axis represents chromosome, and the Y-axis represents the -log10 of the *p*-value of the association test. At a significance threshold of -Log10 (*p*) ≥ 4.0, this study detected a total of 150 SNPs significantly associated with FORR DSI (Figure 3, Supplementary Table S2). These SNPs were concentrated in 41 QTLs, and genes in the 50 kbp region flanking each peak SNP were screened for FORR resistant candidates. To identify the potential functions of these genes, we classified them into various functional groups according to the Gene Ontology database (http://geneontology.org/ accessed on 16 November 2023). Among the candidate genes, 24 genes had no functional annotation and were from protein families with unknown functions. The other 216 genes were related to hormone metabolism, RNA, protein metabolism, plant growth regulation, signal transduction, and stress defense (Supplementary Table S3).

### 2.4. Candidate Gene Association Analysis and Identification

To explore the potential functions of soybean FORR-related genes, we utilized resequencing data from 60 SGAs (30 lines with higher/lower FORR resistance levels) and conducted gene association analysis using the GLM method (MAF > 0.10). A total of 31 SNPs from 6 genes were identified: *Glyma.07G155300* (7 SNPs), *Glyma.09G235800* (7 SNPs), *Glyma.15G268100* (2 SNPs), *Glyma.15G268200* (5 SNPs), *Glyma.15G268300* (7 SNPs), and *Glyma.15G268400* (3 SNPs). The haplotype loci present on these candidate genes were significantly associated with soybean FORR (Table 2, Supplementary Table S4). The haplotype loci present on these candidate genes showed different notable DSI findings.

**Figure 4:**
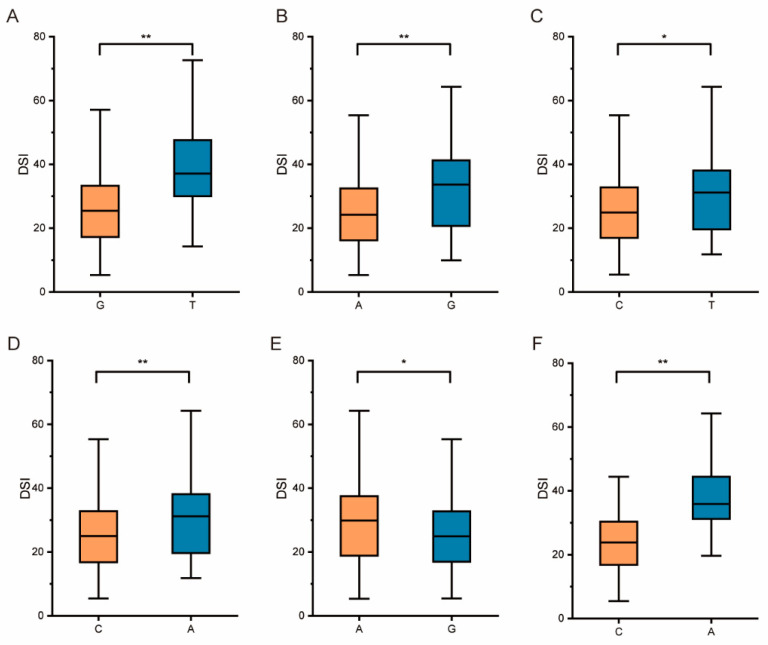
Significant SNP haplotype analysis of FORR DSI across 356 accessions. (**A**) Average DSI of germplasms carrying S07_19078765-G/T; (**B**) average DSI of germplasms carrying S09_45842672-A/G; (**C**) average DSI of germplasms carrying S15_50447372-C/T; (**D**) average DSI of germplasms carrying S15_50452626-C/A; (**E**) average DSI of germplasms carrying S15_50482172-A/G; (**F**) average DSI of germplasms carrying S15_50486939-C/A. * *p* < 0.05, **p < 0.01 (*t*-test).

The allelic variation of S07_19078765 in the candidate gene *Glyma.07G155300* is G/T, which is located in the exon region of the candidate gene. The average DSI of germplasm carrying S07_19077312-G is significantly lower than that of germplasm carrying S07_19077312-T (Figure 4A).

The allelic variation of S09_45842672 in the candidate gene *Glyma.09G235800* is A/G, which is located in the upstream region of the candidate gene. The average DSI of germplasm carrying S09_45842672-A is significantly lower than that of germplasm carrying S09_45842672-G (Figure 4B).

The allelic variation of S15_50447372 in the candidate gene *Glyma.15G268100* is C/T, which is located in the 3′UTR region of the candidate gene. The average DSI of germplasm carrying S15_50447372-C is significantly lower than that of germplasm carrying S15_50447372-T (Figure 4C).

The allelic variation of S15_50452626 in the candidate gene *Glyma.15G268200* is C/A, which is located in the 3′UTR region of the candidate gene. The average DSI of germplasm carrying S15_50452626-C is significantly lower than that of germplasm carrying S15_50452626-A (Figure 4D).

The allelic variation of S15_50482172 in the candidate gene *Glyma.15G268300* is G/A, which is located in the upstream region of the candidate gene. The average DSI of germplasm carrying S15_15:50482172-G is significantly lower than that of germplasm carrying S15_50482172-A (Figure 4E).

The allelic variation of S15_50486939 in the candidate gene *Glyma.15G268400* is C/A, which is located in the 3′UTR region of the candidate gene. The average DSI of germplasm carrying S15_50486939-C is significantly lower than that of germplasm carrying S15_50486939-A (Figure 4F). These allelic variant sites are used for the development of molecular markers.

**Figure 5:**
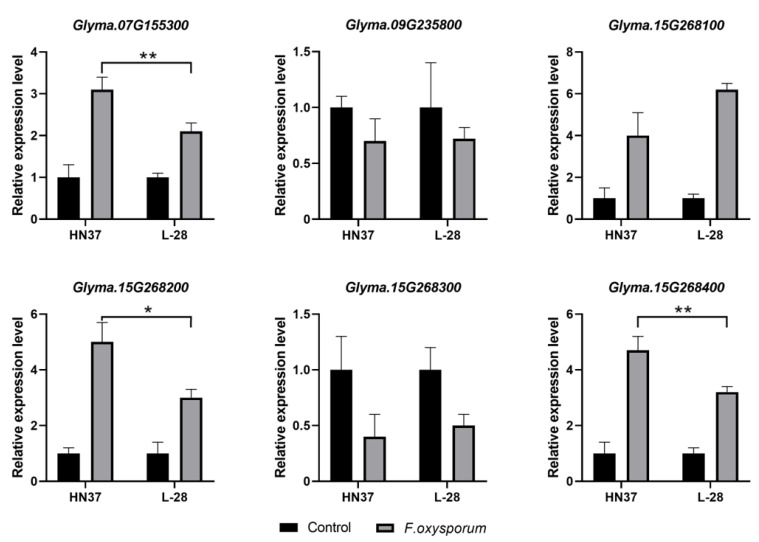
Relative expression level of candidate genes of FORR resistant material HN37 and FORR sensitive material L-28. Values are presented as the means ± SEs (*n* = 3). * *p* < 0.05, ** *p* < 0.01 (*t*-test).

In order to verify the expression levels of candidate genes in disease-resistant materials and sensitive materials, we used HN37 as an extremely disease-resistant material and L-28 as an extremely sensitive material. After treating them with *F. oxysporum*, we took samples at 72 hours post inoculation and performed RT-qPCR. The results showed that after being treated with *F. oxysporum*, the expression levels of some genes in HN37 were higher than those in L-28 (*Glyma.07G155300*, *Glyma.15G268200* and *Glyma.15G268400*). The expression level of *Glyma.15G268100* in HN37 was lower than that in L-28 after *F. oxysporum* stress. In addition to this, there was no significant difference in the expression of some genes between the two materials (*Glyma.09G235800*, *Glyma15g268300*) (Figure 5).

### 2.5. Development of CAPS Markers for FORR Resistance in Soybean

**Figure 6:**
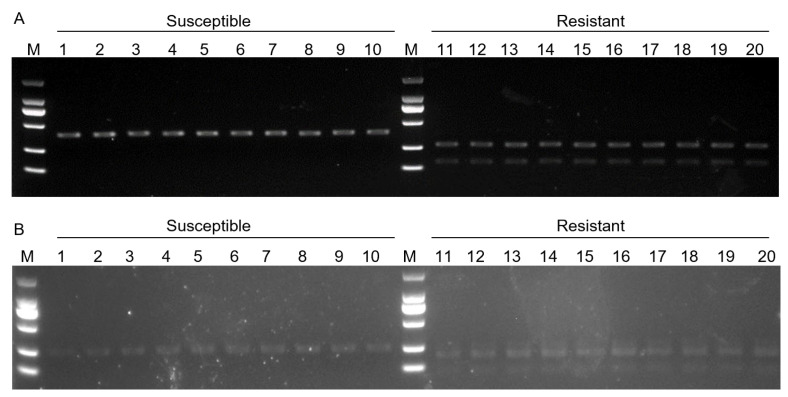
Results of enzyme digestion S15_50486939-CAPS1 and S15_50452626-CAPS2 marker. (**A**) S15_50486939-CAPS1 enzyme digestion electrophoresis. Lanes 1–10 represent FORR-resistant materials, and lanes 11–20 represent FORR-sensitive materials. M. DL2000 Marker (**B**) S15_50452626-CAPS2 enzyme digestion electrophoresis. Lanes 1–10 represent FORR-resistant materials, and lanes 11–20 represent FORR-sensitive materials. M. DL2000 Marker.

Two significant SNPs that associated with FORR resistance, S15_50486939 in *Glyma.15G268400* and S15_50452626 in *Glyma.15G268200*, which covered restriction endonuclease recognition sites GTAC (S15_50486939) (CviQI) and G (S15_50452626) GTCTC (BsaI-HFv2), were suitable to develop CAPS markers. We selected 20 SGAs (10 FORR-resistant varieties and 10 FORR-sensitive varieties) for specific amplification and genotyping of the variant loci. SNP-CAPS marker, S15_50486939-CAPS1 was designed, and C/A alleles were distinguished by CviQI. The digestion products of 359 bp represent the deleterious allele (AA) carried by ten sensitive germplasm resources, while the digestion products of 241 bp and 118 bp represent the homozygous genotype of beneficial allele (CC) carried by ten resistant germplasm resources (Figure 6A). SNP-CAPS marker, S15_50452626-CAPS2 was designed, and C/A alleles were distinguished by BsaI-HFv2. The digestion products of 220 bp represent the deleterious allele (AA) carried by ten sensitive germplasm resources, while the digestion products of 163 bp and 57 bp represent the homozygous genotype of beneficial allele (CC) carried by ten resistant germplasm resources (Figure 6B). Variation in the C/A allele was found to exist in S15_50486939-CAPS1 and S15_50452626-CAPS2 in 20 soybean varieties by sequencing (Supplementary Figure S1). Finally, we successfully developed two CAPS markers, which can be used for the subsequent auxiliary identification of resistance to FORR.

### 2.6. KASP Markers for FORR Resistance in Soybean

**Figure 7:**
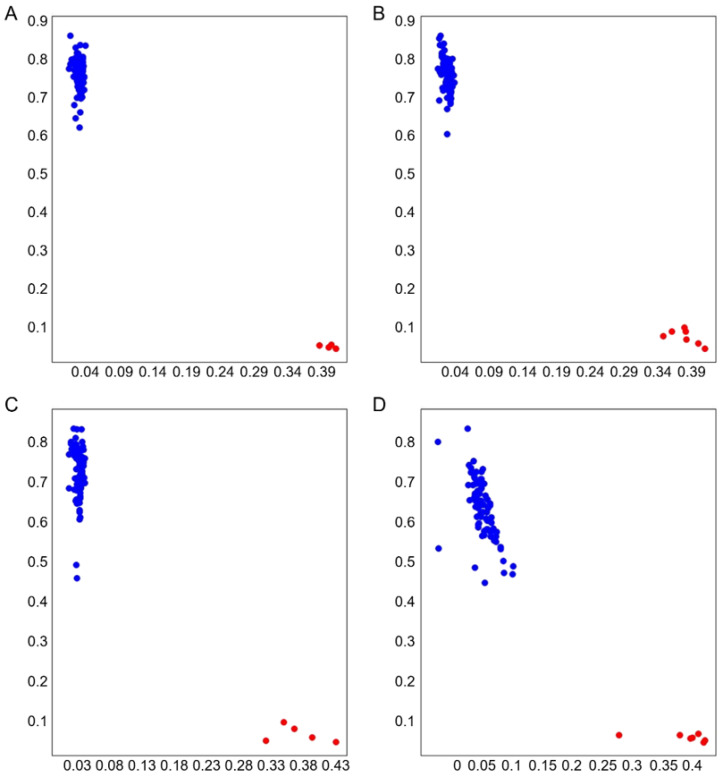
Genotyping of KASP markers. (**A**–**D**) were genotyping of S07_19078765. Blue and red dots represent the soybean germplasm carrying the TT and GG allele mutation sites.

Based on the GWAS results of 356 SGAs, we developed a KASP marker for the locus S07_19078765-G/T of *Glyma.07G155300*, which was significantly associated with the DSI of FORR. The blue dots represent SGAs carrying the TT allele mutation site, indicating a high DSI. In contrast, the red dots represent SGAs carrying the GG allele mutation site, showing the opposite trend. The DSI of soybean accessions with the TT genotype was significantly higher than that of soybean accessions with the GG genotype. The KASP marker developed based on S07_19078765-G/T provides an important reference for further advancing soybean FORR resistance research and breeding (Figure 7A–D).

## 3. Discussion

Soybean root rot is one of the important diseases that leads to reduced soybean yield [1]. Soybean root rot is induced by multiple factors, and the types of pathogens are complex. In soybean production, there are relatively few varieties that are completely resistant to root rot. Simply put, most varieties are only resistant to a single root rot pathogen, which makes it difficult to analyze their resistance mechanism. *F. oxysporum* is ranked fifth among fungal pathogens due to its serious threat to economic crop yields [9]. GWAS is a commonly used and effective method for analyzing important agronomic traits of plants. The discovery of resistance sites will help the genetic improvement in soybean varieties and the discovery of resistance genes, which can improve our understanding of the molecular mechanisms of soybean disease resistance. FORR is a complex quantitative trait. Elucidating the genetic basis and genes associated with FORR is the focus of breeding soybean varieties with durable resistance genes.

Diseases and insect pests have always restricted the yield of soybeans. Researchers in many countries have isolated *F. oxysporum*, a pathogenic bacteria, from various plant diseases including sudden death syndrome, *Fusarium* wilt, and root rot. These plant diseases share a common feature—they infect plants through the root system. As a result, we conducted a phenotypical characterization of root rot caused by 356 SGAs by *F. oxysporum* M38. There are many ways to inoculate *F. oxysporum*, including artificially injected [30], inoculated into the hypocotyl [31], sorghum sand inoculation method [23]. The first and second inoculation methods have the advantage of short preparation time for the inoculum but have the disadvantage of high operational requirements and being prone to inconsistency in wound making. The inoculation method used in this study was similar to the sorghum sand inoculation method, but we used vermiculite instead of sand, mainly because vermiculite can have better air permeability. Moreover, this method has the characteristic of not damaging plant tissues when inoculating plants, which can avoid destroying the structural resistance and chemical resistance of plants and reduce the influencing factors of the experiment. More importantly, greenhouse inoculation is more conducive to controlling variables than field inoculation.

In this study, GWAS analysis was performed to identify QTLs associated with FORR resistance based on resequencing data of 356 SGAs and phenotypes after FORR inoculation. We screened candidate genes based on the functional description and haplotype analysis of the genes within the QTL. We found four candidate genes related to FORR in qFORR-15-4, including *Glyma.15G268100*, *Glyma.15G268200*, *Glyma.15G268300*, and *Glyma.15G268400*. *GmRNF1a* (*Glyma.15g268100*) was identified as a member of the RING family of E3 ubiquitin ligases. Heterologous expression of GmRNF1a in *Arabidopsis* showed that this gene is involved in plant maturation and pod shattering [32]. Meanwhile, *Glyma.15g268100* was also considered as a candidate gene for regulating soybean oil content in the study of Li et al. (2023) [33]. Therefore, this gene may be a pleiotropic gene that is not only related to maturity, pod cracking, and oil content, but also regulates soybean FORR resistance. When inoculated with *F. oxysporum*, the relative expression of *Glyma.15G268200* in FORR disease-resistant materials and susceptible materials changed significantly, indicating that this gene may be involved in the plant′s immune response. The relative expression of this gene in disease-resistant materials at higher expression levels may be related to enhanced plant resistance to *F. oxysporum*. *Glyma.15G268300* contains VQ domain. VQ proteins have been shown to be functional in plant stress and disease resistance studies. Overexpression of *GmVQ35* and *GmVQ47* in *Arabidopsis* resulted in increased susceptibility to *Botrytis cinerea*. Furthermore, transgenic *Arabidopsis* overexpressing *GmVQ47* also showed increased sensitivity to heat [34]. In our study, *Glyma.15G268300* responded to FORR and may have a function in FORR resistance. *Glyma.15G268400* contains the Molybdenum Cofactor Synthesis C domain. The molybdenum cofactor is involved in the synthesis of nitrate reductase, peroxisomal sulfite oxidase, aldehyde oxidase, and xanthine dehydrogenase, which regulate key metabolic processes in plants, thereby playing an essential role in enhancing plant resistance to diseases and environmental stresses [35]. Overexpression of the *Arabidopsis* molybdenum cofactor sulfurylase gene (*LOS5*) in maize increases its aldehyde oxidase activity and improves drought resistance [36]. *LOS5* has also been reported to improve drought resistance in soybean [37]. In this study, *Glyma.15G268400* responded to FORR and had a higher relative expression in disease-resistant varieties, which may be related to its important role in plant disease resistance immune response and may be involved in regulating plant defense mechanisms. We also identified a QTL qFORR-7-2 associated with FORR, which contains the candidate gene *Glyma.07G155300*, which has a pre-mRNA-splicing factor Ntr2 domain. For plant disease resistance, mRNA splicing factors are essential. *AtSKRP* affects plant immune responses to *Phytophthora capsici* by positively and negatively regulating plant immunity through association with the spliceosome component *PRP8* and the splicing factor *SR45* [38]. In the *rs33* mutant, the loss of Serine/Arginine Splicing Factor *RS33* leads to abnormal pre-mRNA splicing of a large number of stress response genes, making the plants highly sensitive to abiotic stress [39]. *Glyma.07G155300* may be involved in regulating the pre-mRNA splicing of soybean stress and immunity-related genes, affecting soybean′s disease resistance and stress resistance. These candidate genes related to FORR disease resistance provide new clues for analyzing the molecular mechanism of soybean resistance to FORR.

Molecular markers of plants have great potential in the agricultural field. CAPS and KASP are two of the molecular markers that are used for MAS of plants with specific traits [40,41]. The CAPS marker of the 3’-UTR of the *Glyma07g03490* was developed in soybean and used for the selection of soybean varieties with traits related to 100-seed weight by Shu et al. (2011) [42]. Similarly, CAPS is also used in soybean disease resistance, such as soybean powdery mildew [29], soybean mosaic virus [25], and soybean *Phytophthora sojae* resistance [28]. In this study, two new FORR resistance-related CAPS molecular markers were developed, namely S15_ 50486939-CAPS1 and S15_50452626-CAPS2. Two CAPS markers can effectively distinguish disease-resistant and disease-susceptible genotypes and can be used in soybean disease-resistant breeding to achieve the purpose of rapid screening of resistant varieties. Recently, KASP has become a cost-effective MAS method and has been applied in the research of stress and disease resistance of various crops, such as wheat stem rust [43], *Pisum sativum* seedborne mosaic virus [44], and Melon Powdery mildew [45]. In soybean research, a KASP associated with resistance to *Fusarium graminearum* was developed by Cheng et al. (2017) [46]. In SCN research, a SCN resistance-related KASP was developed by Tran et al. (2017) [47]. In this study, the KASP marker developed based on the upstream variant site G/T of *Glyma.07G155300* can also accurately identify the genotype associated with FORR. The development of these molecular markers makes large-scale, high-throughput resistance screening possible, which helps to accelerate the breeding process of disease-resistant varieties.

In the future, the molecular markers developed in this study, if integrated into larger-scale breeding programs, could help improve the efficiency of breeding disease-resistant varieties. The pathogens of soybean root rot are diverse. Applying the molecular markers developed in this study to resistance research and breeding strategies for different pathogens can not only improve resistance to *F. oxysporum*, but also facilitate the development of soybean varieties with multiple resistances to cope with complex field disease environments. In addition, the candidate genes related to FORR obtained in this study include *Glyma.07G155300*, *Glyma.15G268100*, *Glyma.15G268200*, *Glyma.15G268300*, and *Glyma.15G268400*. The functions and regulatory mechanisms of these genes need further in-depth study. It may help enhance soybean′s resistance to other biological and abiotic stresses and promote the overall adaptability of soybean. The molecular markers and candidate genes revealed in this study provide a new theoretical basis for the resistance mechanism of soybean root rot. In the future, it will help accelerate the breeding of disease-resistant varieties, improve the stress resistance of crops in complex environments, and promote the sustainable development of global agriculture.

## 4. Materials and Methods

### 4.1. Plant Materials and F. oxysporum Isolates

The 356 SGAs used in this study were derived from the Chinese Soybean Genebank (CNSGB) (https://www.cgris.net, accessed on 16 August 2023) and maintained by the Soybean Institute of Northeast Agricultural University. These soybean varieties come from China, the United States, Germany, Australia, Italy, Japan, and Russia, but most of them come from China. This study was conducted in the greenhouse facilities of Northeast Agricultural University (Harbin, China) in September 2023. The *F. oxysporum* (M38) isolate used in this study was isolated and identified from the roots of diseased soybean plants in soybean field in Mishan, Heilongjiang Province, China [48]. The pathogens used in this study were stored on silica at 4 °C in the dark.

### 4.2. Production of the Inoculum and Inoculation Procedure

*F. oxysporum* was incubated on potato dextrose agar (PDA) at 25 °C in the dark for 1 week. Sterile sorghum grains (*Sorghum bicolor* (L.) Moench) were soaked in distilled water overnight and placed in a 150 mL Erlenmeyer flask (100 g seeds/flask) for autoclaving. Seven-day-old mycelium of *F. oxysporum* was inoculated into sterile Erlenmeyer flasks containing sorghum grains and incubated in the dark for 2 weeks. The Erlenmeyer flasks were shaken for 5 minutes every 24 hours to ensure uniform growth of *F. oxysporum*. Sterilized vermiculite was mixed as a nutrient substrate with the crushed and infected sorghum grains. The infected sorghum grains were mixed with sterilized vermiculite at a ratio of 1:50 (V: V) and stirred thoroughly until completely mixed. Four healthy soybean seeds were planted in seedling boxes (250 mL) filled with a mixture of inoculum and vermiculite. Five pots of each variety were used, and after soybean seedlings emerged, the seedlings were reduced to three plants/box. The experiment was conducted using a randomized complete block design. The greenhouse environment was maintained at 12 h light at 25 °C/12 h dark at 18 °C.

### 4.3. Resistance Evaluation in Greenhouse

The soybean identification evaluation criteria in this study were modified from the method described by Chang et al. (2018) [1]. The resistance of soybean varieties to *F. oxysporum* was evaluated based on the soybean roots 15 days after inoculation. Each plant was evaluated using a rating scale from 0 to 7, where: 0 = no symptoms; the plant can grow normally; 1 = taproot was slightly brown, the lateral roots were growing healthily, and the plant was able to grow normally; 3 = taproot was mostly brown, the lateral roots had brown spots, and the growth of the above-ground part was slightly affected; 5 = taproot was completely brown, the lateral roots had obvious brown spots, and the growth of the above-ground part was severely inhibited; 7 = taproot was dark brown and broken, the lateral roots were black, and the plant was dead or not producing seedlings. In total, 15 plants of each soybean variety were used for disease classification surveys, and DSI data were obtained (Supplementary Figure S2). A disease severity index (DSI) was calculated for each accession using the following formula: DSI = (Σ (Rating value × Number of plants with this rating)/Total number of plants surveyed × 7) × 100. Based on DSI, FORR resistance of SGA can be divided into the following categories: highly resistant (HR, 0 < DSI ≤ 10), medium resistant (MR, 10 ≤ DSI < 20), moderately susceptible (MS, 10 ≤ DSI < 30), susceptible (S, 30 ≤ DSI < 60), or highly susceptible (HS, 60 ≤ DSI < 100).

### 4.4. Genotypic Data

The CTAB method was used in this study to isolate genomic DNA of 356 SGAs from soybean leaves [49]. The quality and concentration of DNA were tested by NanoDrop™ One (Thermo Fisher Scientific, Inc., Waltham, MA, USA), and qualified DNA required OD260/OD280 to be in the range of 1.8-2.0. SNP loci were screened based on the principle of minor genotype frequency (MAF) more than 0.05 and missing rate lower than 0.1. A total of 1,355,930 high-quality SNP loci were obtained and used in this study. SNPs were distributed on all 20 chromosomes of soybean. On average, there were 67,796 SNPs per Chr. The number of SNP markers on each chromosome was uneven, and the chromosome variation rate was high. The average SNP marker density of the whole genome was 874.44 bp/SNP. Chr.19 had the highest SNP density (372.64 bp/SNP), while Chr.12 had the lowest SNP density (1915.71 bp/SNP). (Supplementary Table S5.)

### 4.5. Population Structure Evaluation and Linkage Disequilibrium Analysis

Population structure analysis of the 356 SGAs was performed by principal component analysis (PCA) using the GAPIT version 3 software package [50]. The linkage disequilibrium (LD) between the pairs of SNPs was estimated using squared allele frequency correlations (r^2^) in TASSEL version 3.0n [51]. Only SNPs with a MAF ≥ 0.05 and missing data ≤10% were applied to estimate. In contrast to the GWAS, missing SNP genotypes were not imputed with the major allele prior to LD analysis. The program for candidate gene association analysis included MAF (≥0.05) and the integrity of each SNP (≥80%).

### 4.6. Genome-Wide Association Study

The GWAS was performed using the GAPIT 3.0 package based on the R_4.4.0_ software and the Rstudio (version 2024.04.0+735) software [52]. To prevent false positives in association analysis, a Mixed Linear Model (MLM) was utilized in the GWAS [53]. The significance threshold for association loci was set at -Log10(*p*) ≥ 4.0. SNPs that exceeded this threshold were considered to be significant association loci.

### 4.7. Identification of Candidate Genes and qRT-PCR Assay

Candidate genes were screened from a 100 kb genomic region (50 kbp upstream and 50 kbp downstream) of each significant QTLs, and candidate gene identification and annotation were performed using the soybean reference genome (Wm82.a2.v1, http://www.soybase.org, accessed on 10 May 2024) [54]. From the genome resequencing data, variations in the exonic regions, splice sites, 5’UTR and 3’UTR, intronic regions, upstream and downstream regions of the candidate genes in 30 FORR-resistant and 30 FORR-sensitive varieties. TASSEL version 5.0 [51] used the general linear model (GLM) approach to conduct a gene-based association analysis to detect SNPs or haplotypes related to FORR. Significant SNPs affected the investigated trait when the test statistic reached *p* < 0.05. We used online software (https://bar.utoronto.ca/eplant_soybean/ accessed on 12 May 2024) to analyze the expression levels of genes in different soybean tissues and used the NCBI website to analyze the annotation information of these genes. Through a series of cross-screenings, we obtained candidate genes. 

Samples were taken from primary roots 72 h following inoculation, crushed sterile sorghum grains mixed with sterilized vermiculite were used as controls, and total RNA was extracted with the Easy Pure Plant RNA kit (QUANSHIJIN, Beijing, China). RNA was reverse transcribed using the PrimeScript^TM^ RT Reagent Kit with gDNA Eraser (Takara Bio Inc., Kusatsu, Shiga, Japan). The Primer-Blast tool provided on the NCBI website was used to design qRT-PCR primers (Supplementary Table S6). qRT-PCR analysis was performed using a one-step real-time qRT-PCR kit (Toyobo, Osaka, Japan). The transcript levels were normalized to housekeeping gene actin using the 2^-ΔΔCt^ method. The reaction conditions were as follows: 5 min incubation at 94 °C, followed by 45 cycles of 94 °C for 30 s, 60 °C for 30 s and 72 °C for 40 s. Melting curve analysis was conducted from 55 °C to 100 °C with a final cooling step for 10 min at 72 °C. During the qRT-PCR analysis, three biological repetitions and three technical replicates were performed.

### 4.8. Development of CAPS and KASP Molecular Markers

For CAPS molecular markers. The Soybase (https://legacy.soybase.org/, accessed on 20 May 2024) database was used to obtain the upstream and downstream 500 bp sequences of the candidate genes, and the SnapGene (version 6.0.2) software was used to search for the variant sites and the enzyme cleavage sites containing the variant sites. DNA from 10 FORR-resistant and 10 FORR-sensitive varieties was extracted using the 2×CTAB method. Primers containing sequences of mutation sites and restriction sites were designed using the Primer-Blast tool available on the NCBI website (accessed on 25 May 2024). We used restriction endonucleases CviQI and BsaI-HFv2 to enzymatically cut the DNA fragments of these varieties after PCR and purification, respectively (Supplementary Table S7). The polymorphism was detected by separating the products on a 2% agarose gel.

For KASP molecular markers. The KASP markers of S07_19078765 were specifically identified in three sets of primers (F1, F2, and R) that were used for genotyping (Supplementary Table S8). These primers were created using the Primer-Blast tool that can be found on the NCBI website. The method of 2×CTAB was used to extract genomic DNA. The KASP V4.0 2×Mastermix (LGC Genomics, Teddington, UK) was utilized to amplify the PCR, which was performed in accordance with the reagent instructions with a Quantitative Real-Time PCR System (ABI7500).

## 5. Conclusions

In this study, 356 SGAs resources were used for GWAS to systematically evaluate soybean resistance to FORR, and multiple SNP loci and candidate genes closely related to disease resistance were screened. GWAS analysis identified 150 significantly associated SNPs for 41 QTLs and developed CAPS markers that could be used for genotyping, namely S15_50486939-CAPS1 and S15_50452626-CAPS2. The same KASP molecular marker S07_19078765 was developed in this study. The discovery of these markers and candidate genes provides important genetic resources and technical support for subsequent disease-resistant breeding, which helps accelerate the selection and breeding of disease-resistant soybean varieties and improve breeding efficiency. At the same time, this study reveals the important role of genetic diversity in disease resistance and provides new ideas for soybean breeding and disease prevention.

## Figures and Tables

**Table 1:** Evaluation of resistance to FORR in 356 soybean germplasm resources.

DSI	Type of Reaction	SGAs	Percentage (%)
DSI = 0	Immune, I	0	0.0
0 < DSI ≤ 10	High Resistant, HR	19	5.3
10 < DSI ≤ 20	Medium Resistant, MR	96	27.0
20 < DSI ≤ 30	Medium Susceptible, MS	114	32.0
30 < DSI ≤ 60	Susceptible, S	120	33.7
DSI ≥ 60	High Susceptible, HS	7	2.0

**Table 2:** Candidate genes for significant SNPs associated with FORR resistance.

SNP	Chr.	Position (bp)	Alleles	Candidate Genes	Region
S07_19078765	7	19,078,765	G/T	Glyma.07G155300	upstream
S09_45842672	9	45,842,672	A/G	Glyma.09G235800	upstream
S15_50447372	15	50,447,372	C/T	Glyma.15G268100	3′UTR
S15_50452626	15	50,452,626	C/A	Glyma.15G268200	3′UTR
S15_50482172	15	50,482,172	A/G	Glyma.15G268300	upstream
S15_50486939	15	50,486,939	C/A	Glyma.15G268400	3′UTR

## Data Availability

Data are available in the manuscript and in the Supplementary Materials.

## Supplementary Materials

The following supporting information can be downloaded at: https://www.mdpi.com/article/10.3390/ijms252312573/s1.
