# Supplementary material for: Genome-Wide Association Study and Marker Development for Fusarium Oxysporum Root Rot Resistance in Soybean
Source: Int J Mol Sci. 2024 Nov 22;25(23):12573. doi: 10.3390/ijms252312573 (PMC11640847; doi:10.3390/ijms252312573)
Supplement: Supplementary file 1 [file ijms-25-12573-s001.zip › Supplementary Table and Figure--Updated/Supplementary Figure S1.pdf]

Figure S1. (A)S15_50452626 sequence. (B)S15_50486939 sequence.

(A)

GWAS_040	TTACAAATTGGAGACCATTTCAGCTTT
GWAS_111	TTACAAATTGGAGACCATTTCAGCTTT
GWAS_037	TTACAAATTGGAGACCATTTCAGCTTT
GWAS_113	TTACAAATTGGAGACCATTTCAGCTTT
GWAS_182	TTACAAATTGGAGACCATTTCAGCTTT
GWAS_041	TTACAAATTGGAGACCATTTCAGCTTT
GWAS_215	TTACAAATTGGAGACCATTTCAGCTTT
GWAS_128	TTACAAATTGGAGACCATTTCAGCTTT
GWAS_233	TTACAAATTGGAGACCATTTCAGCTTT
GWAS_213	TTACAAATTGGAGACCATTTCAGCTTT
GWAS_330	TTACAAATTGGAGACAATTTCAGCTTT
GWAS_275	TTACAAATTGGAGACAATTTCAGCTTT
GWAS_177	TTACAAATTGGAGACAATTTCAGCTTT
GWAS_318	TTACAAATTGGAGACAATTTCAGCTTT
GWAS_322	TTACAAATTGGAGACAATTTCAGCTTT
GWAS_094	TTACAAATTGGAGACAATTTCAGCTTT
GWAS_184	TTACAAATTGGAGACAATTTCAGCTTT
GWAS_175	TTACAAATTGGAGACAATTTCAGCTTT
GWAS_165	TTACAAATTGGAGACAATTTCAGCTTT
GWAS_161	TTACAAATTGGAGACAATTTCAGCTTT
Consensus	ttacaaattggagac atttcagcttt

(B)

GWAS_040	ATATGCCTAGGTACAACTGAAAAAA
GWAS_111	ATATGCCTAGGTACAACTGAAAAAA
GWAS_037	ATATGCCTAGGTACAACTGAAAAAA
GWAS_113	ATATGCCTAGGTACAACTGAAAAAA
GWAS_182	ATATGCCTAGGTACAACTGAAAAAA
GWAS_041	ATATGCCTAGGTACAACTGAAAAAA
GWAS_215	ATATGCCTAGGTACAACTGAAAAAA
GWAS_128	ATATGCCTAGGTACAACTGAAAAAA
GWAS_233	ATATGCCTAGGTACAACTGAAAAAA
GWAS_213	ATATGCCTAGGTACAACTGAAAAAA
GWAS_330	ATATGCCTAGGTAAAACTGAAAAAA
GWAS_275	ATATGCCTAGGTAAAACTGAAAAAA
GWAS_177	ATATGCCTAGGTAAAACTGAAAAAA
GWAS_318	ATATGCCTAGGTAAAACTGAAAAAA
GWAS_322	ATATGCCTAGGTAAAACTGAAAAAA
GWAS_094	ATATGCCTAGGTAAAACTGAAAAAA
GWAS_184	ATATGCCTAGGTAAAACTGAAAAAA
GWAS_175	ATATGCCTAGGTAAAACTGAAAAAA
GWAS_165	ATATGCCTAGGTAAAACTGAAAAAA
GWAS_161	ATATGCCTAGGTAAAACTGAAAAAA
Consensus	atatgcctaggta aactgaaaaaa
