# Supplementary figures and images for: Genome-Wide Association Study and Marker Development for Fusarium Oxysporum Root Rot Resistance in Soybean
Source: Int J Mol Sci. 2024 Nov 22;25(23):12573. doi: 10.3390/ijms252312573 (PMC11640847; doi:10.3390/ijms252312573)

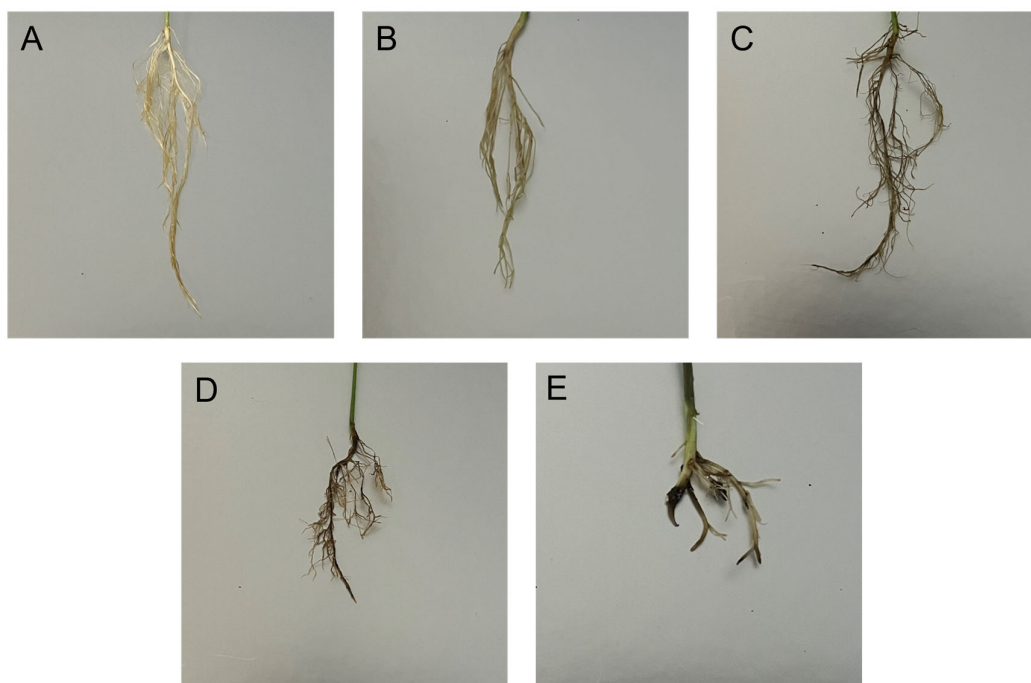

**Figure S2.** FORR resistance scoring standard. A-E are scored as 0, 1, 3, 5, and 7 respectively.

Supplement: Supplementary file 1 [file ijms-25-12573-s001.zip › Supplementary Table and Figure--Updated/Supplementary Figure S2.pdf]
